# Next-Generation Sequencing in the Understanding of Kaposi’s Sarcoma-Associated Herpesvirus (KSHV) Biology

**DOI:** 10.3390/v8040092

**Published:** 2016-03-31

**Authors:** Roxanne Strahan, Timsy Uppal, Subhash C. Verma

**Affiliations:** Department of Microbiology and Immunology, School of Medicine, University of Nevada, 1664 N, Virginia Street, MS 320, Reno, NV 89557, USA; roxcrocks@gmail.com (R.S.); tuppal@medicine.nevada.edu (T.U.)

**Keywords:** Kaposi’s sarcoma-associated herpesvirus, KSHV, next-generation sequencing, genomics, transcriptomics, epigenomics, virology

## Abstract

Non-Sanger-based novel nucleic acid sequencing techniques, referred to as Next-Generation Sequencing (NGS), provide a rapid, reliable, high-throughput, and massively parallel sequencing methodology that has improved our understanding of human cancers and cancer-related viruses. NGS has become a quintessential research tool for more effective characterization of complex viral and host genomes through its ever-expanding repertoire, which consists of whole-genome sequencing, whole-transcriptome sequencing, and whole-epigenome sequencing. These new NGS platforms provide a comprehensive and systematic genome-wide analysis of genomic sequences and a full transcriptional profile at a single nucleotide resolution. When combined, these techniques help unlock the function of novel genes and the related pathways that contribute to the overall viral pathogenesis. Ongoing research in the field of virology endeavors to identify the role of various underlying mechanisms that control the regulation of the herpesvirus biphasic lifecycle in order to discover potential therapeutic targets and treatment strategies. In this review, we have complied the most recent findings about the application of NGS in Kaposi’s sarcoma-associated herpesvirus (KSHV) biology, including identification of novel genomic features and whole-genome KSHV diversities, global gene regulatory network profiling for intricate transcriptome analyses, and surveying of epigenetic marks (DNA methylation, modified histones, and chromatin remodelers) during *de novo*, latent, and productive KSHV infections.

## 1. Introduction

The most recently discovered human tumor virus, Kaposi’s sarcoma-associated herpesvirus (KSHV), or human herpesvirus 8 (HHV-8), is a γ2-lymphotropic-oncogenic virus that causes life-long persistent infection in humans. It is a major cause of AIDS-defining malignancies worldwide [1]. KSHV belongs to the *Rhadinovirus* genus within the *Herpesviridae* family, and it is categorized together with Epstein-Barr virus (EBV), murine gammaherpesvirus-68 (MHV-68), and herpesvirus saimiri (HVS) (reviewed in [2]), with a KSHV numbering system to designate open reading frames (ORFs) based on HVS homology. This human pathogen, initially identified nearly two decades ago from Kaposi’s sarcoma (KS) lesions using the representational difference analysis technique [3], is also associated with two distinct lymphoproliferative disorders: primary effusion lymphoma (PEL), or body cavity-based lymphomas (BCBLs), and multicentric Castleman’s disease (MCD)-linked plasmablastic lymphoma [4,5,6]. KSHV infection still remains a major cause of morbidity and mortality for immunosuppressed individuals, particularly organ transplant recipients and patients infected with human immunodeficiency virus (HIV) [7]. KSHV seroprevalence varies according to geographic region and ethnicity; vial infection is widespread in sub-Saharan Africa and the Amazon basin where more than half of the population is infected [8]. Lower levels of KSHV seroprevalence are reported in Northern Europe and North America with seropositivity ranging from 3% to 10% [9].

The KSHV genome is highly conserved, and it has a high degree of sequence identity across the viral strains. However, two major gene regions, including K1/VIP (a variable immunoreceptor tyrosine-based activation motif protein, encoded by the 5′ terminus of the KSHV genome) and K15/LAMP (a latency-associated membrane protein, encoded by the 3′ terminus of the KSHV genome), located at either ends of the viral genome, are highly variable compared to the central genomic region [10,11]. The sequence variability of the K1 gene has led to the identification of five major KSHV subtypes (A, B, C, D, and E), showing up to 35% variability at the amino acid level across the viral strains [10]. The sequence analysis of the K15 gene has led to the characterization of KSHV sequences, with variants designated as P, M, or N alleles, differing by up to 70% at the amino acid level [11,12]. In addition, nine distinct genomic loci (approximately 5.6% of the genome) contain additional variability, including T0.7/K12, K2, K3, ORF18/19, ORF26, K8, ORF73, and two loci within the ORF75 gene regions, within the central, more conserved region of the KSHV genome [12]. Based on the K1/K15 variability, strain classification, and variability of nine ORFs, the known KSHV genomes are classified into 12 principal genotypes.

Advancement in understanding oncovirus genomes is directly associated with the availability of molecular analytical tools, such as Northern blotting, serial analysis of gene expression, custom oligonucleotide microarrays, real-time polymerase chain reaction (PCR), and nucleic acid sequencing. In the late 1970s, two distinct DNA sequencing methods, based on either chemical cleavage of DNA or incorporation of dideoxy-nucleotides, were simultaneously reported by Gibert *et al.* and Sanger *et al.*, respectively, and are known as first-generation sequencing methods [13,14]. Later, the development of fluorescently labeled dideoxy-nucleotides and automated capillary electrophoresis enabled the use of DNA sequencing or Sanger sequencing technology as part of routine laboratory and medical care [15]. Due to lower error rates, longer-read length (1000 bps obtainable sequence length), and robust performance, Sanger sequencing dominated genomics research and clinical practices until the introduction of new, cost-effective, high-throughput, and high-capacity DNA sequencing platforms were developed to more thoroughly investigate complete genomes and deliver fast, inexpensive, and accurate genetic information.

**Figure 1:**
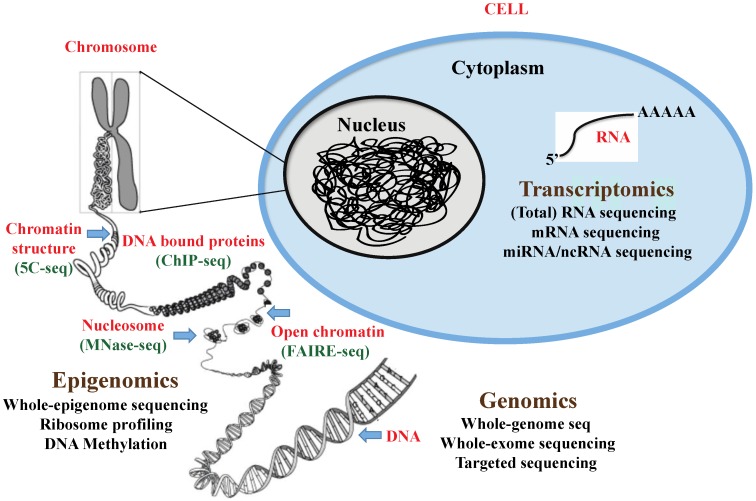
Schematic representation of the different applications of “Next-generation” DNA sequencing technologies.

The novel DNA sequencing methodology, referred to as second-generation sequencing or next-generation sequencing (NGS), has revolutionized the field of cancer genomics and bioinformatics. The major impact of the genome technology revolution is that it has enabled the analysis of cancer genome sequences and the genome structure and the elucidation of mechanisms of cancer pathogenesis, leading to improved tumor diagnosis and treatment. These novel digital NGS methods have provided greater speed, sensitivity, and resolution at a considerably lower per-base cost over other traditional sequencing methods. The arsenal of NGS methods has already been used for whole-genome genotyping, whole-exome mapping, mutation detections, *de novo* assembly, and reassembly of genome, gene-expression profiling, long non-coding RNA profiling, and protein-DNA or protein-RNA binding site detection. The schematic depicting the different methods for NGS studies is presented in Figure 1. This review will summarize the recent scientific findings from the application of high-throughput technologies, and it will discuss the contributions these studies have made to unravel the intricate KSHV genomes. It will also provide valuable insights into the viral regulation of host and viral gene expression profiles during the different modes of KSHV infection.

## 2. Unlocking the KSHV Genome and Insights into KSHV Infection from Host Genomics: The Contributions of Whole-Genome Sequencing, Whole-Exome-Sequencing and Targeted-Sequencing

Whole-genome sequencing or DNA sequencing is used to determine the complete DNA make-up of a genome in order to gain pertinent information about genes, genetic variations, and gene functions. Massive-scale sequencing and analysis of genomic sequences provides a comprehensive characterization of complex genomes, structural variants, and a full spectrum of genomic alterations, such as single-nucleotide substitutions, small insertions and deletions, inversions, translocations, chromosomal rearrangements, and copy number alterations [16]. Recent advances in bioinformatic methods for genomic sequencing have resulted in improved variant detection and increased genomic coverage, due to the powerful combination of high-quality, short-read, and paired-end sequencing options.

The first KSHV genome was mapped in 1996, with phage and cosmid libraries from the lymphoid BC-1 cell line (GenBank ID: U75698.1) using the automated Sanger sequencing approach [17]. That study reported an almost-complete nucleotide sequence of the KSHV genome, except for the sequence for a 3-kb region at the end of the genome that could not be cloned into the sequencing vectors. The KSHV genome is reported to be a large (~165 kb) encapsidated double-stranded (ds) DNA genome consisting of a single 140.5 kb long unique coding region (LUR) with 53.5% guanine-cytosine-rich (G+C-rich) 801 bp long terminal repeat (TR) sequences at both ends of the linear viral genome. The LUR sequence has at least 81 viral open reading frames (vORFs) that encode for polypeptides larger than 100 amino acids, including 66 ORFs with homology to HVS ORFs. The study also identified a duplication of an LUR fragment in the TR region, indicative of genomic rearrangements [17]. One year later, Neipel *et al.* generated a nearly complete sequence of the KSHV genome (GenBank ID: U93872.2) using shotgun sequencing of fragments obtained after partial digestion of DNA extracted from a KS biopsy specimen [18]. The first extensively annotated and fully sequenced KSHV genome, GK18 (GenBank ID: AF148805.2), was obtained from the isolate of KSHV from the biopsy of classic KS lesions from a Greek patient [19].

Since viral mutagenesis is a powerful tool for examining the functions of viral genes, Gao *et al.* generated and sequenced a full-length recombinant KSHV genome, named KSHV BAC36, by inserting a bacterial artificial chromosome (BAC) cassette between ORF18 and ORF19 of the KSHV genome in the BCBL-1 cell line (GenBank ID: HQ404500.1), and they analyzed the function of a number of viral genes [20]. Furthermore, Yakushko *et al.* noted a duplication of a 9-kb LUR fragment in the TR region while sequencing the complete KSHV BAC36 genome [21]. Consequently, Jung *et al.* constructed a new full-length BAC clone of the KSHV genome, called BAC16, derived from the rKSHV.219 virus in JSC-1 PEL cells (GenBank ID: GQ994935.1); complete sequence analysis data indicated a minimal level of sequence variation across the entire viral genome in comparison to the other KSHV strains [22]. All of these KSHV whole-genome sequences were analyzed using the traditional Sanger sequencing. Nevertheless, they are included in this review for completeness.

The most recently sequenced KSHV strain, isolated from cells co-infected with HHV6 and known as DG-1 (GenBank ID: JQ619843.1), represents the first whole-genome KSHV sequence to be completed using Illumina next-generation sequencing of total DNA directly from patient plasma and peripheral blood mononuclear cells (PBMCs), rather than a tissue-cultured or biopsied tumor [23]. A comparison of the whole-genome sequence of this newly discovered KSHV isolate to the KSHV reference sequence (NC_009333) led to the identification of small indels in both the polymorphic regions of K1 and K15 and the TR regions. However, no novel DNA insertions, ORFs, or deletions in the entire coding regions were identified [23].

Very recently, in an effort to correlate KSHV whole-genome diversity and its potential role in KS pathogenesis, genome-scale profiling of KSHV from KS skin lesions of 16 Zambian/sub-Saharan African patients was achieved using Illumina paired-end sequencing [24]. Whole-genome analysis of these KSHV isolates, and multiple alignments of the newly sequenced KSHV genomes, showed a high level of sequence conservation but a low level of variability across the central conserved region. The observed low level variability in the otherwise highly conserved central genomic region indicates that different whole-genome KSHV variants are present in sub-Saharan Africa compared to Western countries, thus resulting in 16 new and distinct Zambian whole-genome phylogenetic structures [24].

In addition, a full genome sequence of the closest homolog of KSHV, retroperitoneal fibromatosis-associated herpesvirus Macaca nemestrina (RFHVMn), was recently analyzed from a clinical KS-like macaque tumor sample using Illumina Genome Analyzer IIx sequencing, indicating a strong genetic and sequence similarity between the two viruses [25]. The RFHVMn genome was annotated based on the KSHV genome, and it was found to have high levels of sequence conservation in specific promoter sequences and within the putative origins of replication. This suggests similarities in the biology and pathology of the two viruses. Another study by Depledge *et al.* used specific-targeted genomic enrichment and whole-genome profiling of 13 herpesvirus genomes, including varicella-zoster virus (VZV), EBV, and KSHV, directly from a range of low volume clinical samples, including blood, saliva, vesicle fluid, cerebrospinal fluid, and tumor cell lines [26]. That study demonstrated that target enrichment technology was suitable for isolating very low quantities of viral DNA from the pool of complex host DNA.

In addition to KSHV genome sequencing, researchers are currently also interested in identifying the host genetic variants that are important to KSHV infection and disease progression via genome-wide association studies. Because the exome represents only approximately 1% of the genome, whole-exome sequencing yields higher sequence coverage and reduces sequencing cost and time by eliminating the majority of the genome from the analysis. Exome-sequencing also facilitates the identification of possible locations or genes for tumor-causing mutations. Using the whole-exome sequencing approach in a single patient, Byun *et al.* identified that development of classic KS in childhood might also occur due to an inborn alteration in a gene that is important for immunity [27]. They revealed a homozygous splice-site mutation in the stromal interaction molecule 1 (STIM1) gene that is very important for immunity, and they identified STIM1 deficiency and the associated primary T-cell immunodeficiency as the genetic cause of severe KS in childhood [27]. In another report, they showed that T-cell-mediated immunity to KSHV is greatly impaired by autosomal recessive complete OX40 protein deficiency, due to R65C (Arg65Cys) loss-of-function mutation in a healthy adult with childhood-onset classic KS [28]. OX40 is a co-stimulatory receptor that is present in activated T-cells [29]. The reported data strongly suggest a causal relationship between OX40 deficiency and the development of KS infection. Another research group reported the whole-genome and whole-exome sequencing-based identification of the signal transducer and activator of the transcription factor 4 (STAT4) gene as a potential classic KS predisposing gene, and they suggested that the variation in the STAT4 gene is linked to KS/HHV-8 infection [30]. In addition to whole-exome sequencing, NGS has also been used to target either a handful of host genes or specific genomic regions to detect cancer-causing mutations. Targeted sequencing allows for rapid production of large numbers of low-cost reads on specific genomic locations at much higher coverage levels over conventional methods. In a recent study, Dittmer *et al.* described the X-chromosome targeted sequencing in 15 PEL cell lines in order to identify tumor-specific single nucleotide variants (SNVs) on the X chromosome [31]. They identified 34 common missense mutations in protein-coding regions in all of the PEL samples, including a Phe196Ser change in the interleukin 1 receptor-associated kinase 1 (IRAK1). This IRAK1 mutant was found to be constitutively phosphorylated and essential for PEL growth and survival. As IRAK1 binding to the MyD88 adapter protein mediates toll-like receptor (TLR) immune signaling, mutation in IRAK1 is considered to be an essential driver for KS-based lymphomas, and inhibition of IRAK1/MyD88 immune signaling might serve as a potential target for drug development [31]. Thus, the combined knowledge of high-throughput genome sequencing and host genetic and genomic variations has provided unprecedented and unbiased insights into viral and host genomes, allowing for the identification of several viral-host factors that influence the progress of KSHV.

## 3. An Atlas of the KSHV Transcriptome: The Contributions of RNA-Sequencing and Small RNA/Non-Coding RNA-Sequencing

### 3.1. RNA-Sequencing

The transcriptome is the complete set of transcripts encompassing a multitude of total coding and non-coding RNAs (ncRNAs), particularly small RNAs, including microRNAs (miRNAs) and ncRNAs, to name a few. These are transcribed in a given cell type under given physiological and pathological conditions. An analysis of the complex landscape and dynamics of the transcriptome enables identification of the full set of transcripts, which reveals the differential gene expression patterns, genome annotations, gene rearrangements, ncRNA detection, and sequence variations that are present at the genomic loci [32]. Initial transcriptomics studies using a hybridization-based microarray and traditional low-throughput sequencing-based approaches used a reference genome, and they allowed a limited dynamic range of detection, had a high background signal, and only provided the ability to detect more abundant transcripts. In contrast, high-throughput NGS, referred to as high-throughput transcriptome sequencing or RNA-sequencing (RNA-seq), provides a new method for mapping and reconstructing the entire transcriptome even without any prior knowledge of the genome [32]. The new methodologies for RNA-seq studies provide enormous sequencing depth (base-pair-level resolution), with a very low background signal, a much higher dynamic range of expression levels, and the ability to identify rare transcripts with regulatory functions [33]. Almost all RNA-seq data are generated through three commercially available NGS platforms: Illumina IG, Roche 454 Life Science, and Applied Biosystems SOLiD. Library preparation for RNA-seq requires a sample of purified, total, or fractionated RNA, which is converted to cDNA fragments with adaptors attached to one or both ends, before sequencing on a NGS platform in order to generate short sequences from one end (single-end sequencing) or both ends (pair-end sequencing) [32]. Targeted RNA sequencing, which focuses on sequencing the messenger RNA (mRNA) and small RNA (ncRNA or miRNA) targets, is achieved by including additional isolation or enrichment steps before cDNA synthesis.

### 3.2. Genome-Wide Analysis of the Latent and Lytic KSHV Transcriptomes

Like all herpesviruses, KSHV undergoes both latent and lytic replication cycles. In a cell culture, primary infection generally results in latency in which only a few promoters that drive the latent RNAs are active, although a small number (1% to 5%) of the latently infected cells spontaneously reactivate the lytic cycle [34,35,36,37]. Viral latent gene expression is critical for inducing and maintaining latency, and it is restricted to the complex multicistronic latency-associated transcript that encodes the viral genes for the latency-associated nuclear antigen (LANA) (ORF73), vCyclin (ORF72), and vFLIP (ORF71), along with a downstream promoter driving the transcription of the viral miRNA cluster and an ORF for the Kaposin (K12) gene [38,39,40,41,42]. After lytic induction using various environmental or chemical inducers [43,44,45,46,47], KSHV transcribes many of the lytic genes that encode the regulatory proteins that are critical for the viral life cycle. Lytic mRNAs of KSHV can be grouped, according to their timing and expression in response to the protein synthesis/DNA replication inhibitors, as immediate early (IE), early (E) and late (L) proteins that encode mRNAs [48,49,50,51]. The IE mRNAs of KSHV include 3.6-kb mRNA encoding RTA/ORF50, 1.7 kb mRNA encoding ORF45, and a 2.0 kb mRNA encoding ORF K4.2 [50]. The latent to lytic switch of KSHV is regulated by the replication and transcription activator (RTA), a 110 kDa protein, encoded by the ORF50 gene, which is capable of inducing the cascade of lytic gene expression. Interestingly, only the over-expression of the RTA protein is necessary and sufficient to disrupt KSHV latency and initiate the lytic replication cascade [52,53,54]. In the lytic cycle, the expression of E transcripts is controlled by the IE transcripts, and it includes polyadenylated nuclear RNA (PAN RNA), Kaposin, ORF57, k-bZIP (K8), K5, K9, K14, K15 ORF6, ORF21, and ORF74 [1], followed by the expression of the L transcripts, including the major capsid protein (MCP) encoded by ORF25 and the small viral capsid (sVCA). Late transcripts are transcribed following DNA replication, and they are the structural genes for virus assembly [48,49].

Transcriptome profiling of KSHV-infected cells, KS lesions, and tissue samples is fundamental to furthering our understanding of the interplay of the large number of underlying mechanisms that coordinate the regulation of KSHV viral gene expression within infected host cells. While the transcription of a few individual genes and promoters of KSHV have been well studied, little is known of the overall landscape of the viral transcriptome on the genome-wide scale [55]. Large-scale gene expression profiling of KSHV transcripts using DNA microarrays, real-time PCR arrays, and Northern blotting have revealed the dynamics and complexity of the viral genome [41,55,56]. Proteomic studies of KSHV-infected cells have assessed the expression of many of the predicted KSHV ORFs [57]. A number of NGS-based research studies have been reported in recent years providing useful insights into the complex KSHV transcriptome, necessary for interpreting the functional elements of the genome and understanding the related KS pathogenesis. During lytic KSHV infection, large segments of the genome that were initially thought to be “non-coding” are actually most likely the stable RNAs [55,58]. Recently, Arias *et al.* analyzed the transcription and translation profiles of the KSHV genome using a combined mRNA-seq ribosome footprinting (Ribo-seq) and genomic DNA sequencing (DNA-seq) approach to uncover new features about the genomic landscape and peptide-coding capacity of KSHV during the productive (lytic) stage of infection when the progeny virion production is underway [58]. Numerous unknown genomic and functional features were assessed using a tightly controlled and highly inducible epithelial iSLK-219 cell line containing a doxycycline inducible transgene that encodes viral RTA protein. This led to the generation of a novel revised annotation of the KSHV genome: KSHV 2.0, including a more than 45% increase in peptide coding potential with expanded illustration of 49 viral transcripts encoding peptides smaller than 100 aa, 63 new ORFs, ncRNAs, polyadenylation sites, splice junctions, and initiation/termination codons of multiple known ORFs (see Table 1) [58]. In addition, the authors of that study attributed the coding capacity of KSHV to multiple strategies, including splicing, mRNA editing, and the use of alternative transcription start sites leading to multiple upstream ORFs (uORFs) and small ORFs (sORFs). The systematic genome-wide survey of the viral transcriptional and translational activity added to our understanding of the underappreciated complexity of KSHV gene expression during distinct phases of the viral life cycle [58]. However, if the new KSHV genome annotation is conserved among multiple cell types that harbor a stably- and latently-maintained KSHV episome still needs to be elucidated.

Immediately after the *de novo* infection, an initial burst of lytic transcripts occurs followed by accumulation of the latent transcripts. Additionally, the KSHV viral particles contain a variety of viral mRNAs and several small RNAs, such as viral and cellular miRNAs, ncRNAs, and unusual, small RNAs (usRNAs) [59,60]. Our lab recently performed an in-depth study of the KSHV transcriptome during *de novo* infection of human PBMCs, CD14^+^ monocytes, and telomerase-immortalized vascular endothelia (TIVE) cells using Illumina RNA-seq methodology [61]. The transcriptome analysis demonstrated that a significant amount of KSHV latent and lytic transcripts accumulated quickly in all three cell types at 4 h post-induction (hpi); the concomitant silencing of latent and lytic transcripts occurred after 24 hpi for CD14^+^ cells and after 48 hpi for PBMCs and TIVE cells [61]. These results are indicative of a cell-type-specific KSHV gene expression pattern that occurs early during KSHV infection. During that period, the viral transcripts that were detected in significant amounts included PAN RNA, ORF58/59, kaposin B, K2, K4, K6, ORF11, ORF17, ORF45, ORF27, ORF37, ORF57, ORF64, ORF65, ORF73, and T0.7. RNA-seq analysis showed that the expression of transcripts involved in lytic DNA replication, such as ORF59, ORF9, ORF6, ORF56, ORF40, ORF54, ORF60, ORF61, ORF70, and ORF37, and transcripts encoding viral structural proteins, such as K8.1 and ORF65, occurred as soon as 4 hpi. The detailed analysis of the RNAs composition in the KSHV virions showed that a large number of viral proteins and mRNAs, including PAN RNA, ORF58, ORF59, T0.7, and ORF17, are encapsidated in the infectious virions that are effectively transduced into the target cells [61]. Furthermore, the Click-iT Nascent RNA Capture Kit was used to differentiate between the RNAs present in the virions and those transcribed during the early stage of infection. The RNA-seq of the newly synthesized RNA captured from the 5-Ethynyl-2′-deoxyuridine (EdU)-labeled KSHV-infected PBMCs showed increased expression of several viral genes that were detected at 4 hpi, including ORF2, K3, T1.5, PAN RNA, ORF26, ORF29, ORF36/37, ORF40, ORF50, K8.1, ORF55, vIRF-2, ORF58/59, ORF60, ORF63/64, ORF67, T0.7, ORF72/73, and ORF74. However, at 24 hpi the active transcription was limited to the ORF73 sequence reads. As expected, the analysis of the transcripts necessary for lytic DNA replication indicated a mixture of the transcripts that were actively transcribed and lytic genes that were transduced with the virions [61].

Several research groups have utilized deep RNA-sequencing approaches to demonstrate the role of various viral and host genes in regulating KSHV gene expression during lytic replication and reactivation. LANA, expressed by KSHV ORF73, is a major latency nuclear protein with pleiotropic functions. It plays an important role in repressing lytic genes by binding to and repressing the ORF50/RTA promoter [62]. It was recently found that LANA recruits the host transcriptional repressor, Krüppel-associated box domain-associated protein 1 (KAP1), and represses the RTA promoter [63,64]. However, the location, mechanism, and the dynamics of LANA/KAP1 binding to the RTA promoter have yet to be investigated. Chandran *et al.* identified the role of the host transcription factor; nuclear respiratory factor 2 (Nrf2) in modulating KSHV gene expression in latently infected PEL-derived cell lines [65]. Cellular Nrf2 plays an important role in the dynamic changes in RTA expression. In the absence of LANA, Nrf2 acts as a transcriptional activator, but it functions as a repressor in the presence of LANA. This switch in the role of Nrf2 on the RTA promoter is mediated by LANA-regulated recruitment of KAP1. Nrf2 inhibition further resulted in increased KSHV lytic gene expression, virion production, and PEL cell death. Collectively, this study demonstrated that KSHV-induced Nrf2 facilitates lytic gene expression during *de novo* infection, and it later represses this induction via LANA-mediated KAP1 recruitment to the Nrf2 binding site [65]. In a study by Swaminathan *et al.*, the role of KSHV ORF57, a lytic-phase KSHV protein that is essential for lytic replication and virion production, was examined using a combination of deep RNA-seq, quantitative reverse transcription polymerase chain reaction (qRT-PCR), Northern blotting, and rapid amplification of cDNA ends (RACE) analysis [66]. To investigate the mechanism by which the ORF57 gene product enhances KSHV gene expression, a comparison of all of the KSHV lytic transcripts in the presence or absence of ORF57 was conducted. The results indicated that two clusters of the KSHV lytic cycle transcripts are expressed more abundantly in the presence of ORF57 [66]. In that study, eight transcripts that were expressed during early and late lytic replication were found to be particularly ORF57-dependent, and they are involved in DNA replication, DNA packaging, virion assembly, and immune modulation. A 2014 study by Gong *et al.* sought to examine the role of previously uncharacterized KSHV ORF18 and ORF30 genes during KSHV *de novo* lytic replication in papilloma-immortalized human oral keratinocyte (HOK16B cells) [67]. Using the KSHV ORF18- and ORF30-deficient virus, high-resolution profiling of transcripts via RNA-seq indicated that these genes are essential for efficient late gene transcription during both *de novo* KSHV infection and lytic reactivation. Studies of late gene transcription in beta- and gammaherpesviruses provide further evidence that ORF18 and ORF30, along with ORF24, ORF31, ORF34, and ORF66 (gammaherpesvirus nomenclature), belong to the virus-specific pre-initiation complex necessary for the expression of late viral genes [68,69,70]. In comparison to high-resolution global examination of KSHV latent and lytic transcripts, relatively fewer NGS-based studies have been conducted to determine the link between the virus and the host mRNAs. In this context, foremost studies by Glaunsinger *et al.* revealed that the SOX (host shutoff and alkaline exonuclease) protein encoded by the KSHV ORF37 protein interacts with host translation machinery and shuts off host gene expression in the early stage of infection by accelerating global mRNA turnover [71,72]. KSHV-induced mRNA turnover is governed by a coordinated destruction by both the KSHV exonuclease SOX and the mammalian 5′–3′ mRNA exonuclease XrnI in a two-step process where the mRNAs are initially cleaved internally by the SOX protein, followed by degradation via cellular XrnI [73]. Notably, the cytoplasmic mRNA-targeting SOX protein has been recently shown to cause cellular mRNA decay in mammalian cells by altering RNA polymerase II (RNAPII) transcription in the nucleus via a feedback mechanism that occurs in response to the catalytic activity of XrnI [74]. High-throughput RNA-seq analysis of SOX-expressing cells has indicated selective destabilization of endogenous cellular p53-induced protein with death domain (PIDD), but not Apoptosis Enhancing Nuclease [75] or interleukin-6 (IL-6) transcripts by SOX. This suggests that cellular mRNAs utilize several different mechanisms to escape SOX-mediated shutoff [76,77]. Cleavage of cellular mRNAs by the KSHV SOX protein is both target- and cleavage-site specific, as defined by the sequence of the target RNAs. In order to shed light on the targeting mechanisms of SOX, Gaglia *et al.* mapped the location of SOX cut sites across the human RNA transcriptome using a degradome sequencing technique, called parallel analysis of RNA ends (PARE), and targeted mutagenesis [78]. The sequences surrounding the SOX cleavage sites in the endogenous mRNA targets were found to have a degenerate sequence pattern that enables SOX to achieve a broad targeting capability while maintaining cut site specificity within the mRNA [78]. Recently, Conrad *et al.* performed a high-throughput sequencing of RNA isolated by crosslinking immunoprecipitation (HITS-CLIP), to analyze ORF57-cellular RNA interactions in KSHV-infected cells during lytic reactivation [79]. They identified ORF57 interactions with both the virus and the host RNAs throughout the KSHV genome and at the KSHV origins of lytic replication.

In addition to studying viral and cellular gene expression profiles during persistent and/or productive KSHV infection, NGS-based transcriptome studies have been recently extended to discover new virus-tumor associations and the underlying mechanism that drives infection and causes virus-mediated oncogenesis. In a recent large-scale investigation of 50 cancer cell lines, Cao *et al.* used high-throughput RNA-seq to detect the presence of different viruses that are known to infect humans/mammalian cells and to study virus-host interactions [80]. Along with other significant discoveries, including the detection of a new unknown murine leukemia virus infection and the identification of cellular cytokine regulators disrupted by virus integration, global assessment of the KSHV transcriptome in the PEL-derived BCP-1 cell line indicated that viral immune signaling genes in the left cluster of the genome, such as K2/vIL-6, K4/vIL-8, and K5/E2-ubiquitin ligase 1, showed higher expression values of 840 FPKMs (fragments per kilobase per million mapped reads), 145 FPKMs, and 351 FPKMs, respectively, when compared to a LANA-containing rightward cluster with an expression value of 39 FPKMs; this suggests that secreted cytokines have a significant impact on both latent and lytic KSHV-infected cells [80].

### 3.3. Small RNA/Non-Coding RNA-Sequencing

#### 3.3.1. Role of miRNAs in KSHV Latency and Lytic Reactivation

Two classes of small (~21–25 nucleotide (nt)) ncRNAs that regulate genes and genomes are: miRNAs and short interfering RNAs (siRNAs) [81]. The miRNAs bind to target messenger RNAs (mRNAs) and cause mRNA translation inhibition or degradation by targeting complimentary sequences in the 3′ untranslated regions (3′ UTR) of the mRNAs [82,83]. These regulatory RNAs play a pivotal role in a variety of biological processes, including viral infection, and dysregulation of miRNAs promotes disease progression and viral cancer pathogenesis [84]. In addition to large numbers of cellular miRNAs, bioinformatics, sequencing, and direct cloning methods have been used to identify and characterize several miRNAs of viral origin [85]. Like all herpesviruses, it is now evident that KSHV encodes many miRNAs in latently and/or productively infected cells (reviewed in detail in [83,84,86,87]). Although only a limited number of protein-coding genes are expressed during latency, KSHV expresses 25 mature miRNAs (at last count) derived from 12 precursor miRNAs (pre-miRNAs) that are located in one cluster in the major latency-associated locus (118–128 kb); it also modulates cellular miRNAs as a means of affecting cell development and cell cycle regulation [88]. The mature KSHV miRNAs are annotated as miR-K1 to miR-K12, depending on their proximity to the Kaposin gene. The KSHV miRNAs are constitutively expressed in the PEL cell lines and de novo infection models during the latent as well as lytic modes of viral infection. The expression levels of KSHV miRNAs *in vitro* is shown to be controlled by both latent and inducible lytic promoters, although the expression levels of these miRNAs during the natural KSHV infection is yet to be identified [40,89,90]. Furthermore, KSHV miRNAs sequences are highly conserved (approximately 99.6%) among several PEL cell lines and KSHV isolates [91].

Eleven of the 12 currently known distinct KSHV miRNAs, miR-K1 to miR-K11, were initially identified by cDNA cloning and traditional sequencing in latently infected B-cells, while miR-K12 was predicted computationally followed by microarray and Northern blot analysis [92]. Sequence analysis using small RNA high-throughput sequencing on the Solexa/Illumina and ABI SOLiD platforms has offered further insights into the miRNA coding potential of KSHV. The first, in-depth analysis of small viral miRNA-expression in the latently KSHV-infected BC-3 cell line by Umbach *et al.* demonstrated that KSHV-encoded miRNAs account for approximately 92% of the total miRNA cDNA reads [93]. Significant discrepancies were revealed between the primary obtained viral sequences for miR-K8 and miR-K12, in comparison to the sequences submitted in miRBase [90,92]. The miR-K9 stem-loop located within an approximately 1.7 kb sequence that represents the origin of lytic genome replication, ori-LytB [94,95], appears to be highly mutated in the KSHV-infected BC-3 cell line, thus affecting the miR-K9 expression. The study also reported the first detection of several KSHV-miRNA-offset RNAs (moRNAs) in mammalian somatic cells, for nine of the 12 KSHV miRNAs expressed from the KSHV latency region, although the moRNAs were not abundant [93]. As reported earlier by Pfeffer *et al.,* two distinct seed sequences, miR-K10a and miR-K10b, were detected for miR-K10; these isoforms differ by one nucleotide due to the differential processing at the 5′ end of miR-K10 [89].

In another study, Lin *et al.* used deep sequencing of small RNA libraries to identify a total of 25 different 5p- (derived from the 5′ arm) and 3p- (derived from the 3′ arm) derivatives of mature KSHV miRNAs obtained from all 12 pre-miRNAs from cells undergoing lytic infection [96]. This study also reported the detection of several novel small viral moRNAs and antisense viral miRNAs (miRNA-AS). KSHV miRNAs, such as miR-K1, miR-K3, miR-K4-3p, miR-K6-3p, and miR-K11 are expressed at detectable levels, unlike the miR-K9 stem-loop, which seemed to have a very low or undetectable expression level; this finding was consistent with earlier reports [86,90,93]. This differential expression of KSHV miRNAs could be attributed to either the differences in the processing and stability of miRNAs or to their incorporation into the RNA-induced-silencing complex (RISC).

Another interesting aspect of KSHV miRNA sequences that might shed light on KSHV virology stems from the observation that several viral and cellular miRNAs as well as usRNAs have been detected in the KSHV virions via Illumina HiSeq 2000 sequencing and *in situ* hybridization-electron microscopy (ISH-EM) [59]. Numerous KSHV miRNAs, and a subset of poorly expressed host miRNAs, are preferentially packaged into KSHV virions during virus production, and they have been hypothesized to play a role in viral maturation [97,98]. In fact, these virional miRNAs are able to enter host cells where they function to repress gene expression during *de novo* infection. Given that almost all of the herpesviruses, polyomaviruses, and adenoviruses have been shown to encode their own miRNAs, it is possible that these DNA virus particles also consist of viral-encoded miRNAs. In addition, a significant number of usRNAs have a sequence that is similar to has-miR-K1246, and these are, possibly, processed from a U2 snRNA or are transcribed from their own promoter in the KSHV virions [59]. It is believed that these virional miRNAs and usRNAs might help the virus evade the host immune system and trigger KSHV latency during *de novo* infection; however, their exact role remains unclear.

Identifying the mRNA targets and functional significance of KSHV miRNAs is critical for understanding KSHV infection and oncogenesis. KSHV miRNAs are known to regulate the viral life cycle, the cell cycle, apoptosis, angiogenesis, and host immune surveillance [99,100]. Recently, ribonomic approaches, such as HITS-CLIP and PAR-CLIP (photoactivatable ribonucleoside-enhanced crosslinking and immunoprecipitation) have been used to identify the positions of protein-KSHV miRNA interactions with a higher resolution and specificity [101,102]. In addition to encoding KSHV miRNAs, KSHV is also known to regulate the expression levels and functions of numerous cellular miRNAs. Using PAR-CLIP, more than 2000 cellular mRNA targets of KSHV miRNAs have been recently identified, including those that are likely to influence KSHV pathogenesis [103]. Moreover, Ago HITS-CLIP, a technique that combines UV cross-linking, immunoprecipitation of Ago-miRNA-mRNA complexes, and deep sequencing, identified 1170 and 950 putative cellular KSHV miRNA targets in two-KSHV-latently-affected PEL cell lines, BCBL-1 and BC-3, respectively [102]. Significant differences between these cell lines were observed in the Ago-association of KSHV and host miRNAs. This suggests that significant differences might exist in their respective miRNA targetomes.

Small RNA-sequencing and poly-A-enriched mRNA sequencing of viral and cellular miRNAs in latently KSHV-infected cells identified differential expression of miRNA-mRNA target pairs [104]. That study revealed 153 differentially expressed human miRNAs, most of which were significantly downregulated, including the large 14q32 miRNA cluster that is shown to be deregulated in human diseases and cancer. Correlation of miRNA expression levels to their respective mRNA target genes using TaqMan assay indicated up-regulation of miR-708-5p, leading to the down-regulation of caspase-2 and leukemia inhibitory factor (LIF), as well as down-regulation of miR-409-5p, a miRNA associated with an increase in the p53-inhibitor MDM2 [104].

The miRNAs that bind to the 3′UTR of the target transcripts play an important role in the posttranslational regulation of a gene expression program [81,82]. The 3′UTR refers to the portion of an mRNA transcript that extends from the stop codon to the polyadenylated tail, and it affects transcript stability, subcellular localization, and translation efficiency [105,106]. To date, KSHV 3′UTRs have been determined for more than 75% of protein-coding RNAs [58,107,108]. In the past few years, several research groups have performed genome-wide mapping and screening of KSHV transcripts and 3′UTRs to identify numerous bicistronic and polycistronic transcripts in several KSHV genomic loci as novel targets of KSHV miRNAs [58,107,109,110]. The bicistronic and polycistronic transcripts have been identified at numerous KSHV genome loci, including ORF30–33, ORF34-37, ORF50-K8, and ORF71-73 [38,50,111,112]. Knowledge of a likely role for KSHV 3′UTRs in the control of the KSHV life cycle has further increased since Sullivan *et al.* suggested that some KSHV 3′UTRs might differentially regulate KSHV gene expression during latent *vs.* lytic infection [107]. Mapping of the 3′UTRome of major KSHV latent and lytic transcripts from TREx BCBL1-RTA cells using Illumina RNA-seq analysis, 3′ rapid amplification of cDNA ends (3′RACE) revealed KSHV transcripts with common polyadenylation sites, leading to shared 3′UTRs and similar 3′UTR-mediated regulation. Approximately half of the KSHV 3′UTRs, including those with higher levels of guanine-cytosine (GC), showed a decrease in gene expression during latent infection. In addition, that study indicated that some KSHV 3′UTRs are sufficient to impart increased gene expression during lytic infection [107]. A study by Gao *et al.* indicated that KSHV miRNAs frequently target the extended 3′UTR of the 5′-proximal ORFs and 5′-distal ORFs of bicistronic and polycistronic transcripts [109]. Screening of the 3′UTR sequences and polyadenylation sites of 74 KSHV genes has identified that 11 out of 28 KSHV miRNAs targets are bicistronic or polycistronic transcripts that are spread across the entire KSHV genome. Reporter mutagenesis suggests that miR-K3 specifically targets the locus that encodes for lytic genes ORF31–33, thereby resulting in the inhibition of their protein expression. On the other hand, the KSHV isoforms miR-K10a-3p and miR-K10b-3p, and their variants, repressed ORF71, ORF72, and ORF73 transcripts in the latent locus through binding sites in the 5′-distal ORFs and intergenic regions. In addition, a variety of KSHV-encoded miRNAs have been shown to directly target some lytic genes, including ORF50 (miR-K9* and miR-K12-7-5p), ORF56/57 (miR-K5 and miR-K6-3p), and ORF-K2 (miR-K10a-3p), and modulate the latent-to-lytic phase transition [102,113,114,115].

#### 3.3.2. Role of ncRNAs in KSHV Lytic Reactivation

The impact of NGS technologies on ncRNAs discovery and characterization is particularly noteworthy. Because this topic has been reviewed extensively elsewhere [116,117], we only provide a brief summary here.

During lytic infection, one of the most abundant viral transcripts present in KSHV-infected cells is a 1.1 kb, long non-coding PAN RNA [118]. Originally called T1.1 or nut-1 RNA, this early lytic gene product plays a major role in the regulation of viral and host transcriptional programs during all phases of the KSHV life cycle [119,120,121,122]. The PAN RNA locus is located between 28,661 and 29,741 nucleotides and between K6 and ORF16 within the KSHV genome [120]. The high expression of PAN RNA is regulated by the lytic switch K-RTA protein that transcriptionally activates PAN RNA by binding to the closely related response elements, and by ORF57 or mRNA transcript accumulation (Mta), which binds and stabilizes PAN RNA from the cellular RNA decay pathways [123,124,125,126]. The ORF57 interaction and stability of PAN RNA depends on the 9-nucleotide core in the Mta-responsive element (MRE-II) in the 5′ PAN that also binds cellular PABPC1 [121,125]. A recent report by Arias *et al.* utilized mRNA-seq, along with Ribo-seq and genomic DNA-seq, to provide further knowledge about the coding potential of PAN RNA. By applying these techniques in parallel, that research group demonstrated that PAN RNA accounts for 78%–90% of the KSHV reads as early as 24 hpi [58]. Interestingly, the Ribo-seq analysis of KSHV-infected cells revealed the presence of initiating and elongating ribosomes on the “non-coding” nuclear PAN RNA that protects the PAN RNA during the lytic cycle; this indicates that either a fraction of PAN RNA is cytoplasmic or ribosomes can access the PAN RNA in the nucleus for translation. Thus, ribosome-bound RNA might regulate mRNA encoding for several putative peptides, which, if stable, could be very abundant [58]. In addition, this study identified three PAN-encoded peptides, PAN1.1, PAN1.2, and PAN1.3, which are indicative of the increased coding capacity of PAN. Rossetto *et al.* used chromatin isolation by RNA purification coupled with next generation sequencing (ChIRP-seq) approach [127], an invaluable tool for mapping specific lncRNA–DNA interactions, and showed that PAN RNA binds and interacts with the KSHV genome to initiate the viral lytic-phase transcription program and occupies much of the KSHV genome during lytic infection, including its own promoter [119]. PAN RNA interacts with specific demethylases, UTX and JMJD3, within the KSHV genome and with the protein components of polycomb repressive complex 2 (PRC2) to globally influence viral and cellular gene expression, respectively [120]. Recently, another non-coding PAN RNA of 3.0 kb (designated as T3.0), transcribed from the opposite strand of the KSHV ORF50, has been identified and reported to encode a small peptide, designated as viral small peptide 1 (vSP-1, 48aa); vSP-1 complexes with RTA proteins and inhibits the proteasome-mediated degradation of RTA, thereby facilitating viral gene expression and lytic replication [128].

## 4. Whole-KSHV Epigenome-Mapping: The Contributions of DNA Methylation-Sequencing, ChIP-Sequencing and FAIRE-Sequencing

The term “epigenome”, meaning “sequence-independent heritable changes of the genome”, refers to a set of reversible biochemical modifications made to both DNA and associated histone proteins, *i.e.*, the building blocks of nucleosomes that do not involve a change in the underlying nucleotide sequence [129]. Examples of these alterations, generally referred to as epigenetic marks or modifications, include DNA methylation, histone modifications, chromatin remodeling, nucleosome occupancy, and coding and non-coding RNA expression, which collectively govern the gene regulatory network involved in several biological processes [130,131,132,133]. In terms of mapping the epigenetic marks and several DNA–protein interactions, a systematic profiling of the epigenomes in various cell types and stages can advance our understanding of how chromatin modifications are patterned genome-wide, and it can provide a clearer picture of transcriptional gene regulation [134].

Tremendous progress in NGS-based technologies has enabled the reproducible assessment of the viral and cellular epigenetic marks across the genome of multiple cell types. Since NGS allows for the unbiased, accurate, high-resolution, and substantially improved sequencing of global and specialized DNA regions, it is considered to be a cost-effective screening tool to evaluate the epigenetic architecture of the KSHV genome in comparison to traditional sequencing approaches. A more precise mapping of protein-binding sites, transcription factors, enhancers, histone variants, and nucleosome positions in the KSHV epigenome has been achieved using a variety of novel sequencing-based assays for profiling of methylated DNA (MeDIP-sequencing and MBD-sequencing) [135], the characterization of DNA-binding proteins (ChIP-sequencing) [136,137], the sequencing of accessible chromatin, via formaldehyde-assisted isolation of regulatory elements (FAIRE-seq) [138], and the discovery of positioned nucleosomes (MNase-seq), at a single-base resolution with the goal of providing new insights into many KSHV-associated diseases.

DNA methylation refers to the reversible methylation of the fifth carbon atom of cytosine in 5′–C–phosphate–G–3′ (CpG) dinucleotides by methyltransferases [139]. Additionally, 5-hydroxymethylation of cytosine (5hmC), catalyzed by the ten-eleven translocation (TET) family of proteins, has also been reported for mammalian cells [140]. Cytosine methylation is believed to play an important role in epigenetic gene regulation, and it is linked to gene silencing by either interfering with the binding of transcription factors or by modifying the chromatin structure [141]. The following assays are commonly used to identify methylated CpG dinucleotides in a genome: methylated DNA immunoprecipitation coupled with sequencing (MeDIP-seq) and methyl CpG-binding domain (MBD) protein sequencing (MBD-seq). These techniques involve capturing methylated DNA fragments using an antibody raised against 5-methylcytosine, followed by library construction and massive parallel sequencing of those fragments to identify the genomic regions of DNA methylation [142]. Histone marks are frequently investigated by chromatin immunoprecipitation followed by sequencing (ChIP-seq), a technique for characterizing and assaying various DNA-binding proteins [134]. In ChIP-seq, the DNA associated with a specific histone modification is immunoprecipitated (using an antibody specific to the protein of interest), and it is used to generate sequencing libraries for genome-wide profiling by NGS [134]. In addition, the chromosome conformation capture (3C) method, a high-throughput technique used to study the interactions between DNA sequences on the same chromosome or between different chromosomes, has been further developed from its original configuration into the carbon-copy chromosome conformation capture (5C) method, which allows for parallel analysis of physical interactions among different chromosomal loci and higher order chromosomal architecture in a high-throughput manner [143,144]. Another important technique, FAIRE-seq, has been coupled with NGS to identify regions of nucleosome-depleted DNA that are generally associated with active transcription [145]. On the other hand, because nucleosome-associated DNA is not affected by microccocal nuclease (MNase) digestion, it is characterized through MNAse-seq to precisely reveal the position and occupancy of nucleosomes in the functional regulatory regions of chromatin [146]. In recent years, scientific researchers have exploited these genomic assays to generate high-resolution epigenome maps to explain the epigenetics involved in KSHV-associated malignancies. In this section of the review, we briefly describe recent genome-wide elucidations of a broad array of chromatin phenomena that have already begun to change our view of the extent and complexity of KSHV epigenomes and their impact on persistent and productive KSHV infections.

### 4.1. Sequencing of the KSHV Epigenome during *de Novo* Infection and Latency

As with other gammaherpesviruses, KSHV virions encapsulate linear double-stranded DNA (dsDNA) in an epigenetically naive configuration without any histones or 5′-methyl cytosine residues. [147] However, upon *de novo* infection, when the virions enter the target cells, the linear dsDNA is circularized to generate a closed-circular DNA, which is maintained as a circular episome and tightly packed as a nucleosome in the infected host cells. The circular episome is further chromatinized for the regulation of KSHV’s biphasic lifecycle, and it is greatly influenced by the same chromatin modulation mechanisms as cellular chromatin. Thus, the chromatinization of the KSHV genome is the key regulatory step for the establishment of tightly repressed quiescent or latent infection.

Multiple insightful studies have uncovered the different spatiotemporal epigenetic changes that occur on the KSHV genome during pre-latency, latency, and lytic reactivation in distinct cell types (reviewed in [147,148]. During the pre-latency or early stages of KSHV infection in endothelial cells, *i.e.*, within 1–8 hpi, a transcriptionally active chromatin, referred to as euchromatin, is present on the KSHV genome, and it is characterized by enhanced levels of the H3K4me3 and H3K27ac histone marks, accompanied by a temporary induction of a few IE lytic genes, including the RTA promoter [135]. However, at 24–72 hpi, the KSHV genome undergoes a transition to heterochromatin, during which the levels of the activating histone marks and the transient expression of the lytic genes decline, with a concomitant increase in the levels of the H3K27me3/H2AK119ub repressive histone marks on the KSHV genome. This facilitates the establishment of latency [135,149]. Thus, the RTA promoter exhibits a bivalent promoter structure, defined by the simultaneous presence of the H3K4me3 and H3K27me3 regions, and it regulates the different stages of the KSHV life cycle. In addition, during the early stages of *de novo* KSHV infection of PBMCs, the K8, ORF49, and ORF64 gene promoters have also been shown to have bivalent chromatin domains [150]. In contrast, the KSHV genome displays a transcriptionally permissive chromatin form in oral epithelial cells during primary infection that results in prolonged and robust lytic gene expression [149]. KSHV latency is also governed by other epigenetic alterations, such as cytosine 5-methylation of the CpG islands and positioning of the nucleosomes on the viral episome [151]. Recently, it has been shown that the KSHV genome in latently infected cells is heavily CpG-methylated across the entire genome, except for the LANA promoter region [135].

Dittmer *et al.* performed FAIRE-Seq to analyze the open chromatin organization and patterns of nucleosome depletion in KSHV latently infected B-lymphoma and epithelial cell lines with a stable and latently maintained KSHV epigenome during latency [138]. They found that approximately 8% of the KSHV genome was associated with the open chromatin regions, while a majority of the viral promoters and genes had closed-chromatin conformation. In general, the quiescent transcription regions or open chromatin regions identified by FAIRE-seq were devoid of nucleosomes and enriched in activating histone marks (H3K4me3 and H3K9/K14-ac). The nucleosome depletion patterns were found to be largely conserved among the latently-infected cells with CTCF-binding sites, coinciding with many nucleosome-depleted loci, excluding the LANA, vIL6, and proximal Kaposin promoter regions, thus, suggesting a common viral genome landscape in all forms of latency [138].

During latency, KSHV maintains a tight control of viral gene transcription, and only a few viral genes that are essential to latent DNA replication and host immune modulations are expressed. Among the KSHV latency genes, the master regulator of latent phase, LANA, which has been reviewed extensively elsewhere [152,153,154], is consistently expressed very early after *de novo* infection in various KSHV-harboring cells and tissues. LANA is crucial for maintaining the KSHV episome by direct and indirect DNA-binding of LANA to the host and viral chromosome [155,156]. Lu *et al.* performed two independent LANA-specific ChIP-seq experiments on latently infected BCBL-1 cells, and they identified a total of 256 LANA-binding sites on the host genome [137]. As might be expected, LANA predominantly binds at the TR region of the KSHV genome, especially at the high-affinity LANA-binding sites, *i.e.*, LBS1/2, and indirectly to the LANA promoter. Notably, some of the LANA-binding sites on the cellular genome were identified as the transcription start sites (TSSs) of numerous genes that are associated with the p53 pathway, and the proximal promoter regions of the genes linked to tumor necrosis factor (TNF) network, both of which are pivotal for maintenance of the KSHV genome [137]. Very recently, another research group investigated LANA binding to multiple active viral and cellular genes by performing a detailed ChIP-seq analysis for LANA, polymerase II (Pol II), and histone marks, *i.e.*, H3K4me3 and H3K27me3 occupancy on the KSHV epigenome in BCBL-1 cells [157]. They also proved that LANA binds to chromatin near TSSs for selected viral genes, confirming earlier published data [136,137]. Promoters for LANA, vIRF-3, and vIL-6a-c displayed a correlation between enrichment of activating H3K4me3 marks and Pol II occupancy (but they were depleted for repressive H3K27me3 marks). Instead, H3K27me3 enrichment was primarily observed in heterochromatic regions, such as lytic late gene promoters, and it was comparatively enhanced in latently infected TIVE (TIVE-LTC) cells in comparison to BCBL-1 cells [157]. The coexistence of LANA with H3K4me3 marks on active host gene promoters prompted researchers to investigate the interaction of LANA with H3K4me3 methyltransferases. The results suggest that LANA associates with H3K4me3 methyltransferase hSET1 complex to create activating histone marks, and it prevents H3K27me3 silencing of latency-associated genes to maintain latency [157].

Integrative analysis of ChIP-seq and RNA-seq data provides a deeper understanding of the interplay between the KSHV’s transcriptome and the epigenome. Recently, Mercier *et al.* used a combined ChIP-seq and RNA-seq approach to study the global relationship between LANA’s occupancy on the cellular and viral genome and its impact on KSHV-driven host gene expression during latency [136]. They found that LANA preferentially binds the host genes near TSSs, and it is highly abundant within transcriptionally active promoters with a sequence identical to the LBS1 motif of KSHV DNA. LANA-bound promoters are enriched in H3K4me3 histone marks, and only a small percentage of the latent genes are differentially regulated in KSHV-infected cells, suggesting that direct LANA-promoter binding is not solely responsible for the altered host gene expression [136]. They also examined the levels of chromatin-bound LANA during lytic induction and found that the binding of LANA to the host and viral chromatin is severely disrupted after 72 hpi in cells undergoing lytic reactivation. This is possibly due to the remodeling of chromatin structure, although the exact mechanism is yet to be identified [136].

As previously mentioned, there is a short period of lytic gene expression during the early stages of KSHV primary infection [158]. Lytic gene expression and the expression of KSHV-encoded LANA protein are quickly shut down, once again suggesting that LANA plays an important role in the establishment of latency. Additionally, LANA is known to down-regulate RTA expression by repressing the RTA promoter [63,159,160]. Two independent research groups recently identified a host protein, KAP1 as a new regulator of KSHV latency that binds to LANA and a significant number of lytic promoters during latency [161,162]. A study by Sun *et al.* demonstrated the plausible mechanism of transcriptional repression of the RTA promoter by LANA-recruited KAP1, during *de novo* infection [63]. Genome-wide mapping of the KSHV genome using ChIP-seq detected multiple co-occupation sites for LANA and host KAP1 on the whole KSHV genome, indicating a direct LANA-KAP1 interaction. The results suggest that LANA interacts with both the N- and C-terminal domains of KAP1 in the nucleus, and it recruits KAP1 to the KSHV genome in order to shut down the lytic gene expression and facilitate the establishment of KSHV latency [63].

### 4.2. Sequencing of the KSHV and Host Epigenomes during Lytic Reactivation and Replication

Several physiological and environmental factors can occasionally trigger the KSHV to transition from the latent to the lytic phase of replication, followed by a complex ordered expression of lytic genes and the release of infectious virus [163]. This dynamic and ordered pattern of viral gene expression is shown to be temporally and epigenetically regulated [135]. The KSHV IE-lytic protein ORF50/RTA functions as the master regulator of KSHV lytic reactivation [53,54,164]. RTA activates the transcription of numerous downstream target genes, either by direct sequence-specific binding to the RTA-responsive elements or by indirect protein–protein interactions with cellular transcriptional factors [165,166,167]. Using genome-wide KSHV genomic microarrays, two independent research groups identified a set of 19 RTA-binding sites located in the promoter, intron, or exon regions in the KSHV genome [168,169]. Two studies have characterized the global presence of histone modifications during reactivation using ChIP-on-chip tiling arrays; they found that activating marks, such as H3K9ac and H3K4me3, are enriched at several loci, including the latency-associated region and the RTA gene promoters, during *de novo* infection, while repressive H3K9me3 and H3K27me3 marks are developed on the RTA promoter, following primary infection [135,170]. A bivalent chromatin structure of an RTA promoter signifies the complexity of the reactivation process that is controlled by a delicate balance between these activating and repressive histone marks. Upon reactivation, PAN RNA recruits H3K4me3 histone methyltransferase and H3K27 histone demethylases to release transcriptionally repressive marks and up-regulate RTA expression [120,171]. In addition, through association with cellular transcription factor CBF1, RTA binds to its own promoter and recruits histone acetyltransferases to further remodel chromatin, thus favoring reactivation [170].

Host epigenetic regulatory mechanisms are also significant for controlling KSHV lytic reactivation. Thus, high-resolution NGS of a host epigenome is fundamental for studying the epigenetics of virus-host interactions. Several host proteins that regulate chromatin organization and RNA transcription bind KSHV genomes at specific sites and play an important role in controlling viral reactivation [172,173]. CTCF and Rad21 have recently been investigated as potent host cell restriction factors that inhibit KSHV lytic transcription [174]. These proteins are known to bind at distinct sites on herpesvirus genomes during latency, although their exact role in lytic induction is still unknown. In an attempt to unravel the mechanisms of viral gene regulation by host proteins, CTCF, and cohesion during KSHV replication, Li *et al.* conducted high-resolution ChIP-seq experiments and examined CTCF and cohesin binding to the viral chromatin. CTCF and cohesin act as global negative regulators of transcription, as their dissociation from the viral chromatin during reactivation leads to a 100-fold increase in virus yield [174]. According to the RNA-seq data, these proteins appear to initially exert an activating effect on transcription; however, their prolonged presence significantly inhibits KSHV lytic transcription and the accumulation of viral RNA. Deep sequencing of host epigenome further indicates that KSHV directly or indirectly modulates the host cell’s Small Ubiquitin-like Modifier (SUMO) system in order to overcome the host’s antiviral immune surveillance that limits KSHV lytic reactivation [175]. SUMOylation is a reversible post-translational modification, and three major SUMO protein isoforms—SUMO-1, SUMO-2, and SUMO-3—have been associated with the epigenetic transcriptional regulation of the chromatin states [176]. Initial studies have identified KSHV-encoded K-bZIP as an enzyme with SUMO E3 ligase activity and specificity towards SUMO-2 and SUMO-3 [177]. Further studies by Chang *et al.* investigated differential genome-wide SUMOylation patterns to identify the role of chromatin SUMOylation during KSHV reactivation [175]. SUMO-1-specific or SUMO-2/3-specific ChIP-seq data has revealed SUMO-2/3 enrichment at the cellular promoter regions of highly transcribed genes and higher K-bZIP-binding on SUMO-2/3 enriched regions. Interestingly, most of the SUMO-2/3-enriched target cellular genes are involved in cell growth, apoptosis, immune response, and cytokine signaling [175]. These studies indicated that SUMO-2/3 marks control the consistent expression of host immune response genes during lytic reactivation. The study was recently extended to demonstrate SUMO-1 and SUMO-2/3 specific chromatin enrichment on KSHV genome during the productive reactivation using chromatin immune-precipitation in conjunction with deep sequencing. An inverse correlation between the SUMO-2/3 marks and the H3K9me3 histone marks was identified. A significant increase in the virus production in SUMO-2/3 knockdown or inactivated viral K-bZIP mutant during reactivation also suggested that SUMO-2/3 modifications on KSHV chromatin could negatively regulate KSHV lytic gene expression and viral reactivation [178].

## 5. Conclusions

The advent of massively parallel sequencing technologies that are still in the early stages of use have revolutionized the field of KSHV genomics, allowing for new biological insights on genomic, transcriptomic, and epigenomic variations. Although sequencing costs have fallen dramatically, NGS data analysis could be difficult in species with complex genomes that are hundreds of kilobases long and contain direct or inverted repeats. This poses significant challenges during the process of sequence assembly. So far, most herpesvirus genome sequencing data have been collected using large amounts of DNA obtained through culturing the virus in permissive cell lines. In uncultured or unenriched samples, a relatively lower abundance of viral DNA than host DNA leads to a lower number of viral reads; thus, it is difficult to assemble the insufficient viral reads into accurate contiguous sequences (contigs) using *de novo* algorithms. As a result, only a few reported sequence characterizations of KSHV genomes are available in the public databases.

Though RNA-Seq can give an unbiased view of KSHV transcriptome without the prior knowledge about each sequence of a transcript, the intricate transcriptome is always more complicated than predicted. Thus, analysis of RNA-Seq data is not a straightforward task, as we still do not know the whole potential of the viral transcriptome. For instance, there might be many viral transcripts that have not been annotated yet. Several other issues that complicate the accurate gene expression profiling using RNA-Seq, include, the presence of multiple related sequences within the KSHV genome, overlapping transcripts from different genes and precise estimation of abundance of different transcripts from the same gene. Further complicating the matter, the current existing databases (e.g., Ensembl) used to interpret RNA-Seq data use the outdated genomic assemblies, hg19 and mm9 (for human and mouse, respectively) and gene annotations (general feature format; GFF files) via the popular pipeline for RNA-Seq analysis TopHat and Cufflinks. This is problematic as significantly increased number of total mapped reads can be captured from each RNA-Seq data with the newest genomic assembly hg38, to give a comprehensive view of transcriptome. Nevertheless, thanks to the rapidly decreasing cost of sequencing, higher resolution and unbiased genome-wide characterization of KSHV genomes can be achieved at a single base-pair scale. Thus, in coming years, if the third generation sequencing technologies that are currently being developed deliver as promised, extensive sequencing data collection using user-friendly and robust software tools will continue to provide laboratory scientists and bioinformaticians with a wealth of additional information to unravel the three-dimensional (3D) KSHV genome for multiple applications.

## Figures and Tables

**Table 1:** New viral features in the KSHV genome including, Upstream and Small ORFs, Alternate start codon usage and Internal ORFs, derived from Next-generation sequencing studies adapted from KSHV 2.0 [57]. All annotation coordinates are based on GQ 994935.1.

Gene	Strand	Start–Stop	Small Open Reading Frame (sORF)	Upstream Open Reading Frame (uORF)	New Features
ORF6.1	+	3027–3203	-	uORF	-
ORF6.2	+	3150–3203	-	uORF	-
ORF10.1	+	14,451–14,531	-	uORF	-
ORF10.2	+	15,574–15,756	-	-	Internal ORF
ORF11.1	+	15,633–15,722	-	uORF	-
ORF11.2	+	15,648–15,722	-	uORF	-
ORF11.3	+	15,693–15,722	-	uORF	-
ORF11.4	+	15,745–15,756	-	uORF	-
ORF11.5	+	15,926–15,991	-	-	Internal ORF
vIL6.6	−	17,873–17,862	-	uORF	-
vIL6.5	−	17,915–17,877	-	uORF	-
vIL6.4	−	18,003–17,902	-	uORF	-
vIL6.3	−	18,047–17,985	-	uORF	-
vIL6.2	−	18,086–18,057	-	uORF	-
vIL6.1	−	18,116–18,057	-	uORF	-
ORFK3A	−	19,128–18,589	-	-	Internal ORF
ORF70A	−	21,099–20,038	-	-	Alternate start
ORFK4A	−	21,820–21,743	-	uORF	-
ORFK4.1a	−	22,517–22,416	sORF	-	-
ORFK4.1d	−	22,610–22,545	sORF	-	-
ORFK4.1e	−	22,653–22,627	sORF	-	-
ORFK4.1c	−	22,806–22,723	sORF	-	-
ORFK4.1b	−	22,850–22,545	sORF	-	-
1.4KbB	+	24,871–24,915	sORF	-	-
1.4KbC	+	24,921–25,058	sORF	-	-
ORFK5.1	−	26,569–26,555	-	uORF	-
ORFK6.1	−	27,647–27,615	-	uORF	-
ORFK6A	−	27,443–27,087	-	-	Alternate start
ORFK6B	−	27,422–27,087	-	-	Alternate start
PAN1.1	+	28,655–28,768	sORF	-	-
PAN1.2	+	28,831–28,965	sORF	-	-
PAN1.3	+	28,888–28,965	sORF	-	-
ORF20A	−	35,322–34,429	-	-	Internal ORF
ORF20B	−	35,202–34,429	-	-	Internal ORF
ORF21.1	+	35,151–35,177	-	uORF	-
ORF25.1	+	42,345–42,380	-	uORF	-
ORF28.1	+	48,758–48,811	-	uORF	-
ORF30.1	+	50,317–50,358	-	uORF	-
ORF34.1	+	54,399–54,485	-	uORF	-
ORF35.1	+	55,419–55,445	-	uORF	-
ORF35.2	+	55,442–55,474	-	uORF	-
ORF37.1	+	57,040–57,126	-	uORF	-
ORF38.1	+	58,251–58,259	-	uORF	-
ORF38.2	+	58,455–58,589	-	uORF	-
43.1-AS	+	63,214–63,228	sORFs	-	-
ORF43.2-AS	+	63,254–63,295	sORFs	-	-
ORF45.1	−	68,447–68,364	-	uORF	-
ORF49.1	−	72,425–72,384	-	uORF	-
ORF50AS	−	74,222–74,130	sORF	-	-
k8.1 short	+	75,890–75,971	-	-	Internal ORF
ORF54A	+	77,552–78,439	-	-	Alternate start
ORF55.1	−	79,501–79,340	-	uORF	-
ORF57A	+	81,464–83,453	-	-	Splice variant
ORF61.2	−	100,071–100,018	-	uORF	-
ORF61.1	−	100,095–100,018	-	uORF	-
ORF62B	−	101,019–100,018	-	-	Alternate start
ORF62A	−	101,061–100,018	-	-	Alternate start
ORF65.1	−	112,321–112,289	-	uORF	-
ORF69.1	+	116,043–116,138	-	uORF	-
Kaposin C2	−	119,084–117,738	-	-	Alternate start
ORF72.1	−	124,182–124,108	-	uORF	-
ORF75.1	−	134,809–134,729	-	uORF	-
ORF75.2	−	134,894–134,817	-	uORF	-
K15.1	−	135,938–135,846	sORF	-	-
